# Editorial: Materials, design, modeling and control of soft robotic artificial muscles

**DOI:** 10.3389/frobt.2022.1074549

**Published:** 2022-11-30

**Authors:** Xiaonan Huang, Andrew P. Sabelhaus, M. Khalid Jawed, Lihua Jin, Jun Zou, Yuzhen Chen

**Affiliations:** ^1^ Robotics Department, University of Michigan, Ann Arbor, MI, United States; ^2^ Department of Mechanical Engineering and Division of Systems Engineering, Boston University, Boston, MA, United States; ^3^ Department of Mechanical and Aerospace Engineering, University of California, Los Angeles, Los Angeles, CA, United States; ^4^ State Key Laboratory of Fluid Power and Mechatronic Systems, Zhejiang University, Hangzhou, China; ^5^ Department of Mechanical Engineering, Fudan University, Shanghai, China

**Keywords:** soft multifunctional materials, soft actuator design, soft actuator modeling, soft actuator control, artificial muscles, soft robotics

## Introduction

Soft robots represent an emerging class of bio-inspired machines mainly composed of soft matter, such as elastomers, fabrics, and fluids. Due to their inherent hyperelasticity and compliance, soft robots can adapt to uneven surfaces and distribute external forces. Therefore, they have potential advantages over traditional piecewise rigid robots in maneuvering through complicated environments.

As the cornerstone of a soft robot, soft robotic artificial muscles have been developed for actuation with various mechanisms, including fluidic actuation, electrical actuation, magnetic field actuation, and light actuation. Despite dramatic advancements in soft robotic artificial muscles in the past decades, many challenges remain in the development of a soft artificial muscle for robots that is capable of swift and precise actuation, produces sufficient forces for dynamic and versatile locomotion, can be controlled with high fidelity, and can be modeled efficiently and accurately. Progress has been limited by the following critical gaps: 1) advanced functional materials in combination with material architectures that can deliver the required actuation performance; 2) mechanical designs that lower the requirement of bulky hardware for untethered operation; 3) physical understanding of the underlying nonlinear mechanics and dynamics of soft artificial muscles for control purposes; and 4) feedback control systems that connect the materials, designs, and models for practical operation of soft robots with artificial muscles.

**FIGURE 1:**
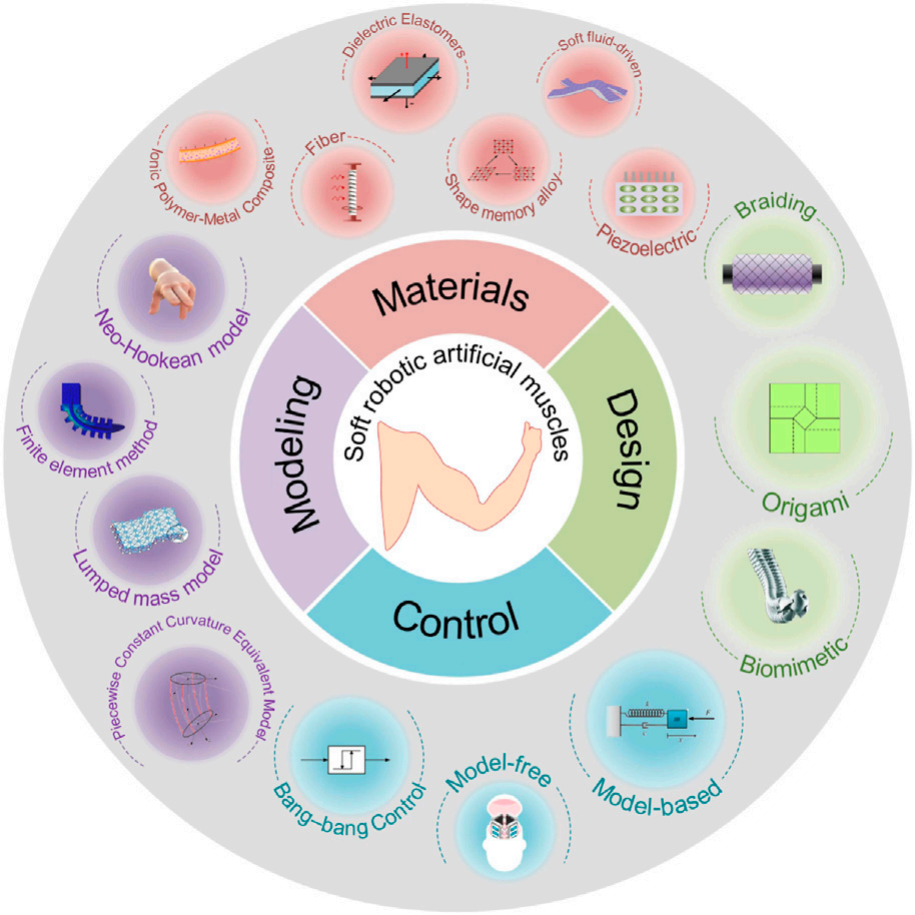
The objective of this Research Topic is to present recent progress on the materials, design, modeling and control of soft robotic artificial muscles.

The goal of this Research Topic is to present recent progress on the materials (e.g., dielectric elastomer and ionic and polymer-metal composite), design (e.g., origami and biomimetic design), modeling (e.g., finite element method and lumped mass model), and control (e.g., model-based and model-free control) of soft artificial muscles that have the potential to advance the performance and robustness of soft robotic motion (Figure 1). The issue includes five contributions that cover the topics of materials, design, modeling, sensing, and control of soft actuators and their application in wearable electronics, underwater soft robots, and mobile soft robots. All five research articles highlight the integration of sensing and actuation into soft matter. The hypothesis and theory article proposes another interesting concept inspired by the octopus: implementing distributed control architecture on soft robots.

Developing highly compliant pressure sensors seamlessly integrating into fluidic-driven actuators and wearable electronics remains a significant challenge. Kang et al. developed a high-permittivity and high-homogeneity elastomer that can be used as elastomeric dielectric layers in pressure sensors. The pressure sensors constructed using this composite elastomer are highly flexible, sensitive, and can sustain at least 100 cyclic deformations without fatigue. The proposed elastomers incorporate imidazole-based ionic liquids and chloropropyl silicone oil into the PDMS. The ionic liquids significantly enhance the relative dielectric permittivity, while the silicone oil promotes overall compatibility, making the elastomer a highly suitable dielectric material. As a result, the relative dielectric permittivity of the proposed elastomer is 105 times higher than that of PDMS. In addition, a very low Young’s modulus (0.78 MPa) and high stretchability (around 321%) are achieved. The proposed high-permittivity elastomers have tremendous potential for the design of wearable electronic devices used in electronic skin applications.

Embodying sensing and actuation capabilities into materials can greatly simplify centralized control and miniaturize the robot’s size. Gosden et al. developed soft bubble artificial muscles that can change their contraction in response to salinity. The artificial muscles are composed of a superabsorbent sodium polyacrylate polymer gel encapsulated in a series of discrete cells made of a heat-sealed Nylon-paper composite. The polymers can swell to over 300 times their initial volume in fresh water. The design of the bubble artificial muscle further translates the volumetric changes of the gels into linear contractions. The characterization of the soft bubble artificial muscles shows a maximum generated force of 10.96 N, a maximum contraction of 27.5% at 1 N load, and repeated actuation and relaxation cycles. This environmentally adaptive artificial muscle opens up new design space and has the potential for underwater soft robots.

The difficulty of modeling electrothermal soft actuators prevents their practical use in soft robots that require precise control due to complex electrical-thermal-mechanical interactions and hysteresis. Sabelhaus et al. tightly integrated compact temperature and bending sensors onto a soft electrothermally-actuated shape memory alloy (SMA) robot limb and developed a framework for its dynamics modeling and control. The framework consists of a neural network based on long short-term memory (LSTM) that takes sensor data as input and outputs the predicted bending angle of a soft limb. The performance is validated in model predictions *versus* a hardware prototpe. The accurate prediction indicates that the proposed framework can capture the core physics that governs these electrothermally-actuated soft limbs. Such framework, therefore, brings hysteretic, difficult-to-model electrothermal actuators (e.g., LCE actuators) into practical soft robotics applications.

Studies on static and dynamic bending properties of Extensile Fluidic Artificial Muscles (EFAM) under various internal pressure can present insights into the design, control, and integration of EFAMs into soft robots. Garbulinski et al. characterized the bending stiffness, natural frequency, and damping ratio of an EFAM experimentally. The bending stiffness was then validated both analytically and numerically using an Euler-Bernoulli beam model and a Planar Discrete Elastic Rod model, respectively. The bending stiffness, damping value and natural frequency increase by 400%, 450%, and 20% as the pressure varies from 25 to 100 psi, 5 to 100 psi, and 30 to 10 psi, respectively.

Although octopuses are intelligent animals, their brains, interestingly, do not do much “computation.” Instead, the brain delegates the tasks to the arms, and the arms can communicate among themselves independent of the brain. Inspired by this, Sivitilli et al. suggested that three control strategies of the octopus can inform and improve the current field of robotics. First is “hierarchical hybrid action selection,” whereby the octopus selects actions by employing a hierarchical structure. This reduces the size of the state space and simplifies the computational task. The second strategy is “ascending recruitment” - this is the concept of one sucker, upon encountering a stimulus, recruiting nearby unoriented suckers. The third and last one is “contact-based control,” which is used to simplify the computational challenge of avoiding collisions in the vast configuration space of the octopus. The octopus relies on contact due to sucker recruitment, which reduces the configuration space and simplifies the control scheme.

Overall, this Research Topic presents novel insights regarding materials, design, modeling, sensing, and control of soft actuators and the robots that use them. The concepts of “integration of sensing and actuation into soft matter” and “implementing distributed control architecture on soft robots” could become two revolutionary trends in the field of soft robotics that can significantly reduce the complexity and size of the hardware design and pave the way for achieving fully autonomous soft robotic systems.

